# Mechanisms of Dietary Sodium-Induced Impairments in Endothelial Function and Potential Countermeasures

**DOI:** 10.3390/nu13010270

**Published:** 2021-01-19

**Authors:** Jordan C. Patik, Shannon L. Lennon, William B. Farquhar, David G. Edwards

**Affiliations:** Department of Kinesiology and Applied Physiology, University of Delaware, 540 S College Ave., Newark, DE 19713, USA; jpatik@udel.edu (J.C.P.); slennon@udel.edu (S.L.L.); wbf@udel.edu (W.B.F.)

**Keywords:** dietary sodium, high salt, nitric oxide, endothelium oxidative stress, glycocalyx, potassium, aerobic exercise

## Abstract

Despite decades of efforts to reduce sodium intake, excess dietary sodium remains commonplace, and contributes to increased cardiovascular morbidity and mortality independent of its effects on blood pressure. An increasing amount of research suggests that high-sodium diets lead to reduced nitric oxide-mediated endothelial function, even in the absence of a change in blood pressure. As endothelial dysfunction is an early step in the progression of cardiovascular diseases, the endothelium presents a target for interventions aimed at reducing the impact of excess dietary sodium. In this review, we briefly define endothelial function and present the literature demonstrating that excess dietary sodium results in impaired endothelial function. We then discuss the mechanisms through which sodium impairs the endothelium, including increased reactive oxygen species, decreased intrinsic antioxidant defenses, endothelial cell stiffening, and damage to the endothelial glycocalyx. Finally, we present selected research findings suggesting that aerobic exercise or increased intake of dietary potassium may counteract the deleterious vascular effects of a high-sodium diet.

## 1. Introduction

Over 90% of adults in the United States consume high-salt diets, with an average daily sodium intake of 3600 mg, which far exceeds the 2300 mg/day as recommended in the Dietary Guidelines for Americans [1,2]. Globally, excess dietary sodium is a contributing factor to 1.65 million deaths annually, with 40% of these deaths occurring before the age of 70 [3]. As such, the consequences of high-sodium diets remain an important topic of study.

The deleterious effects of excess dietary sodium are commonly attributed to the effect of sodium on blood pressure (BP). Importantly, a positive linear association between BP and 24-h urinary sodium excretion, the gold standard marker of sodium intake, has been noted in numerous epidemiological studies [4,5]. Likewise, sodium restriction results in reduced BP in individuals with elevated BP [6,7]. However, among young adults, the magnitude of change in systolic and diastolic BP associated with changes in dietary sodium consumption is a rather modest 1.9 and 0.4 mmHg per 2300 mg/day change in sodium, respectively, after adjustment for BMI and alcohol consumption [5]. In fact, the majority of normotensive individuals are considered salt-resistant in that they do not experience a marked change in BP with large changes in sodium intake [8], especially those under the age of 45 [9].

Yet, even in the absence of elevated BP, high dietary sodium is associated with negative impacts on cardiovascular health. Increased sodium intake is related to increased risk of acute coronary events and cardiovascular mortality independent of BP [10]. Likewise, overweight individuals in the highest quartile of sodium intake have an increased risk of congestive heart failure compared to those in the lowest quartile, even after controlling for demographics, alcohol consumption, smoking, physical activity, and BP [11]. Additionally, greater sodium intake is related to greater left ventricular mass in both normotensive and hypertensive individuals [12,13], and left ventricular hypertrophy is reversed with sodium reduction [14]. Notably, endothelial dysfunction often precedes the development of cardiovascular disease (CVD) [15,16,17] and thus may be an early marker of the deleterious effects of excessive dietary sodium consumption.

**Figure 1:**
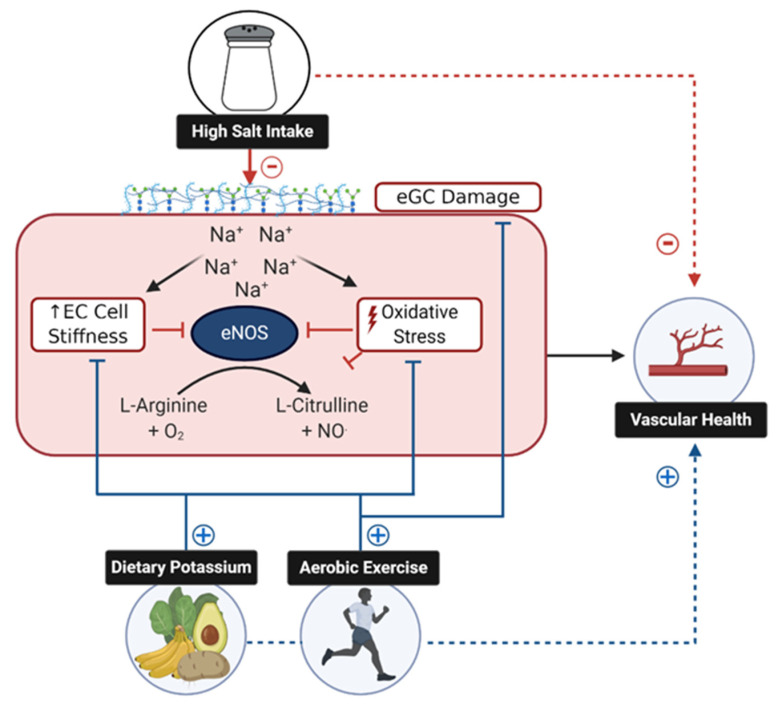
Excess dietary sodium negatively (−) influences nitric oxide (NO)-mediated endothelial function via increases in oxidative stress, an increase in endothelial cell (EC) stiffness, and damage to the endothelial glycocalyx (eGC). Each of these factors results in decreased bioavailability of NO derived from endothelial nitric oxide synthase (eNOS). Evidence suggests that aerobic exercise and/or increased intake of dietary potassium positively (+) influence these factors and thus may be effective strategies to counteract the impact of excess dietary sodium.

As outlined in Figure 1, this review presents the current evidence for BP-independent effects of high dietary sodium consumption on endothelial function in humans, and summarizes the literature examining the potential mechanisms responsible for this phenomenon. Additionally, recent reports suggest that the vascular consequences of excess sodium may be mitigated by physical activity or increased dietary potassium consumption. As such, we will provide a brief review of how these behaviors may address specific mechanisms of dietary sodium-induced endothelial dysfunction.

## 2. Assessment of Endothelial Function

**Figure 2:**
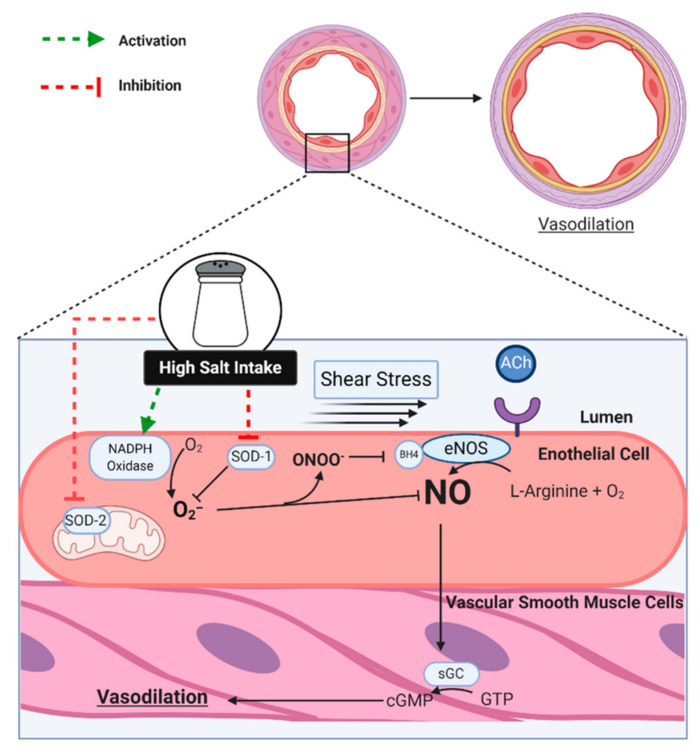
In response to various physiological and pharmacological stimuli, such as shear stress and acetylcholine (ACh), the endothelium produces nitric oxide (NO) via endothelial nitric oxide synthase (eNOS) that diffuses into the vascular smooth muscle cells, where it activates soluble guanylate cyclase (sGC), which then converts guanosine triphosphate (GTP) into cyclic guanosine monophosphate (cGMP), ultimately leading to vasodilation. Excess dietary sodium increases superoxide (O_2_^−^) via activation of nicotinamide adenine dinucleotide phosphate (NADPH) oxidase, while also inhibiting cytosolic superoxide dismutase (SOD-1) and mitochondrial SOD (SOD-2). NO readily reacts with O_2_^−^, thus rendering it unable to diffuse into the VSM. The resulting peroxynitrite (ONOO^−^) oxidizes tetrahydrobiopterin (BH4), which leads to uncoupling of eNOS, leading to further reductions in NO and increases in O_2_^−^ (not shown).

The endothelium is the monolayer of cells lining the lumen of the vasculature throughout the body. Beyond a simple barrier between the blood and the vascular wall, a healthy endothelium serves to prevent thrombosis and coagulation and control vascular tone [18]. In response to chemical or mechanical stimuli, the endothelium produces vasodilating or vasoconstricting substances that act on the adjacent vascular smooth muscle (VSM) cells [18] (Figure 2). One such substance is endothelium-derived nitric oxide (NO), which is frequently studied due to its anti-atherogenic effects, in addition to NO being a key mediator of vascular tone via its potent vasodilating effects [19].

In response to binding of agonists (e.g., acetylcholine or ACh) to endothelium receptors or an increase in shear stress on the endothelium, nitric oxide (NO) is produced when endothelial nitric oxide synthase (eNOS) catalyzes the conversion of oxygen and L-arginine into L-citrulline and NO (Figure 2) [19,20]. The gaseous NO then diffuses into the VSM, initiating an intracellular cascade and causing the removal of cytosolic calcium and/or cellular hyperpolarization, thus producing the relaxation of the VSM and the dilation of the vessel [19,20]. This response can be assessed in vitro in cultured cells and isolated vessels or in vivo in animals and humans. Intra-arterial infusions of ACh or methacholine (MCh) are utilized to test resistance artery endothelial function, and can be used in combination with an NOS inhibitor, such as L-N^G^-Nitro arginine methyl ester (L-NAME) and N^G^-Monomethyl-L-arginine (L-NMMA), to quantify the NO component of the dilation. Ultrasonic assessment of flow-mediated dilation (FMD) of a major conduit artery (i.e., brachial artery) has become commonplace due to its non-invasive nature and the availability of sophisticated analysis software. During the assessment of FMD, the artery in question is imaged via ultrasound before and after a 5-min period of circulatory arrest via a cuff applied to the limb distal to imaging and inflated to a suprasystolic pressure. The increases in blood flow that occur following cuff release increase shear stress on the arterial endothelium, resulting in dilation that is largely NO-mediated [21]. Importantly, impaired endothelium-dependent dilation of the peripheral vasculature is associated with increased risk of future cardiovascular events, and this relation exists whether endothelial function is assessed via FMD [22] or ACh infusion [23,24].

While the studies utilizing FMD demonstrate the effects of excess sodium on the macro-vascular endothelium, the cutaneous circulation can be used as a model of the microvasculature in humans [25]. For instance, a high-sodium diet reduces the NO-mediated increase in skin blood flow in response to local heating in young, salt-resistant normotensive individuals [26]. In agreement with this finding, a high-sodium diet blunts the cutaneous blood flow response to ACh delivered via intradermal infusion [27] or iontophoresis [28,29]. Likewise, cutaneous post-occlusive reactive hyperemia is blunted in the absence of a change in BP following excess sodium consumption [28,30]; however, cutaneous reactive hyperemia appears to be mediated by endothelial release of prostaglandins rather than NO [30,31], and is beyond the scope of this review.

## 3. Dietary Sodium and Impaired Endothelial Function in Humans

A deleterious vascular effect of high dietary sodium in the absence of a change in BP was first noted in the Dahl salt-resistant rat model in 1987 [32]. Subsequent studies, however, suggested that this effect might not occur in humans, as no effect of a short-term high-sodium diet was observed on endothelium-dependent forearm blood flow responses in a small group of young men [33] or middle-aged hypertensives [34,35]. In contrast, Tzemos and colleagues studied young, normotensive adults and found that a five-day high-sodium diet impaired forearm dilation to intra-arterial ACh infusions [36]. When on the high-sodium diet, subjects demonstrated less of an attenuation in ACh-induced dilation by L-NMMA, suggesting that high-sodium diets specifically inhibit NO-mediated dilation [36]. Notably, systolic BP increased on the high-sodium diet, suggesting that a significant proportion of the subjects were salt-sensitive [36], leaving the possibility that the change in BP was responsible for the impairment of endothelial function.

Later studies attempted to eliminate the potential confounding effects of BP. Jablonski et al. observed an improvement in both ACh-induced forearm blood flow and brachial artery FMD after statistically controlling for changes in BP following a four-week reduction in dietary sodium (~1500 vs. 3600 mg/day) amongst middle-aged and older adults with elevated BP [7]. Similarly, DuPont and colleagues observed a significantly blunted brachial artery FMD in young, normotensive, salt-resistant subjects on a high-sodium diet compared to a low-sodium condition [37]. In a subset of subjects, nitroglycerin induced similar dilation of the brachial artery across both diets, providing evidence that the impairment occurred at the endothelium [37] and not the VSM cells. High dietary sodium-induced reductions in FMD have been found in multiple [30,38] but not all [27] studies. There is some evidence that the impact of a high-sodium diet on FMD is greater in men than women [39]. Though these studies typically administer the excess sodium for five to eight days, one research team has demonstrated that FMD is attenuated acutely after a single high-sodium meal [40], highlighting the rapid effect of excess sodium on the vasculature, though this is not a universal finding [41,42]. Table 1 summarizes the details of relevant studies on the endothelial effects of high-sodium diets in humans.

## 4. Mechanisms Contributing to Dietary Sodium-Induced Impairment in Endothelial Function

### 4.1. Oxidative Stress

Excess oxidative stress is the most well-studied mechanistic explanation for dietary sodium-induced endothelial dysfunction. Oxidative stress occurs due to an imbalance between endogenous antioxidant activity and production of reactive oxygen species (ROS), particularly superoxide (O_2_^−^), which results in impaired endothelial function by decreasing NO bioavailability [45]. NO readily reacts with O_2_^−^ to produce the free radical peroxynitrite (ONOO^−^). O_2_^−^ and ONOO^−^ can then oxidize the eNOS cofactor tetrahydrobiopterin (BH4), resulting in the uncoupling of eNOS and generation of O_2_^−^ rather than NO [46,47]. Lenda and colleagues provided early evidence of ROS involvement in high-sodium-induced endothelial dysfunction with the observation that dilation of skeletal muscle arterioles in rats fed a high-sodium diet was restored in the presence of the O_2_^−^ scavenger superoxide dismutase (SOD) [48]. Zhu and colleagues later demonstrated that aortas of normotensive rats fed a high-sodium diet for three days produced less NO and more O_2_^−^ when stimulated by MCh, whereas Tempol, a SOD mimetic, decreased O_2_^−^ production and increased NO [49].

Human studies support a role for oxidative stress in dietary sodium-induced impairments in endothelial function, as outlined in Figure 2. In middle-aged adults with elevated BP who habitually consume a high-sodium diet, ACh-induced forearm blood flow and brachial artery FMD were each augmented following ascorbic acid infusion [7]. Likewise, Greaney et al. demonstrated that local infusion of ascorbic acid restored the NO-mediated increase in skin blood flow to local heating in normotensive, salt-resistant individuals consuming excess sodium [26]. Oral supplementation with the antioxidant vitamins C and E during a high-sodium diet (5500 mg/day) similarly restored the skin blood flow response to cutaneous ACh iontophoresis while also preventing an increase in markers of oxidative stress in the plasma and urine [28].

Within endothelial cells, nicotinamide adenine dinucleotide phosphate (NADPH) oxidase, xanthine oxidase, uncoupled eNOS, and mitochondria can produce O_2_^−^. The origin of O_2_^−^ induced by excess dietary sodium has been investigated by multiple laboratories and/or may vary by species and/or the vascular bed studied. For instance, significantly greater mRNA expression of NADPH oxidase subunits has been reported in the renal cortex of rats fed high-sodium for one week [50]. Likewise, NADPH oxidase and xanthine oxidase activity were increased in the skeletal muscle arterioles of rats fed high-sodium chow, however, inhibition of these enzymes did not restore endothelium-dependent relaxation [51]. Since O_2_^−^ scavenging restores dilation in the skeletal muscle microvasculature [48], the authors speculated that uncoupled eNOS secondary to ONOO^−^ oxidation of BH4 is the source of O_2_^−^ [51]. Similarly, O_2_^−^ production in the skeletal muscle arterioles of high sodium fed mice was decreased in the presence of both Tempol and L-NMMA [52]. The follow-up study by Nurkiewicz et al. confirmed that mice fed high-sodium were BH4 deficient relative to mice on a normal sodium diet, and that this difference and the resulting attenuation in NO-mediated dilation were restored by the addition of L-arginine to the drinking water [53]. Together, these findings suggest that uncoupled eNOS, due to a deficiency of BH4 or L-Arginine, is a source of sodium-induced increases in O_2_^−^.

Zhu and colleagues found that apocynin, an inhibitor of NADPH oxidase, restored dilation in mesenteric arteries of rats fed high-sodium [54]. Additionally, inhibition of eNOS did not decrease O_2_^−^ production in the high-sodium rats, suggesting that uncoupled eNOS was not the source of O_2_^−^ in the mesenteric artery [54]. Likewise, Guers et al. reported that dietary sodium markedly increased NAPDH oxidase expression in rat femoral arteries [55]. Two recent reports demonstrated that apocynin mitigates sodium-associated reductions in cutaneous microvascular dilation to local heating [44] and ACh infusion [27], suggesting that excess dietary sodium increases NADPH oxidase activity in normotensive, salt-resistant humans. Whether this occurs in other vascular beds in humans has not been determined.

In addition to increased production of O_2_^−^, it appears that high-sodium diets may decrease endogenous antioxidant defenses. During inhibition of the cytosolic isoform of SOD, copper-zinc SOD (CuZn SOD or SOD-1), the skeletal muscle microcirculation of rats on a normal sodium diet showed a greater increase in O_2_^−^ than the microcirculation in rats fed a high-sodium diet, suggesting attenuated activity of SOD-1 in the high-sodium condition [56]. Notably, in this study, both groups of rats displayed similar SOD expression and similar responses to catalase inhibition, suggesting that activity of SOD-1 was decreased in the high-sodium condition. Likewise, sodium decreased SOD-1 expression in the femoral arteries of high-sodium fed versus normal chow fed rats [55]. Rats consuming a high-sodium diet also exhibited decreased expression of SOD-1 [57,58] and the mitochondrial isoform, manganese SOD (MnSOD or SOD-2) [57], in the middle cerebral artery; however, this effect may be vascular bed dependent, as the same laboratory did not observe an effect of sodium consumption on either isoform of SOD in the mesenteric arteries [59]. To date, whether vascular expression or activity of SOD are decreased due to dietary sodium has not been directly tested in humans; however, such an effect may partly explain the Tempol-induced increases in NO-mediated cutaneous microvascular function in participants on a high-sodium diet [27,44].

### 4.2. Endothelial Cell Stiffening

In addition to oxidative stress, there is evidence that high dietary sodium intake may decrease NO release via a change in the mechanical properties of endothelial cells. Using cultured endothelial cells, Oberleithner and colleagues report that an increase in the sodium content of the bath, within the physiological range for humans, results in stiffening of endothelial cells, as measured by atomic force microscopy, and attenuated NO release [60]. This only occurred when cells were treated with physiological concentrations of aldosterone, and the stiffening was prevented by amiloride, indicating that opening of epithelial sodium channels (ENaC) induces sodium entry and subsequent cell swelling. Importantly, changes in endothelial cell deformability precede changes in shear-induced NO release, whereas inhibition of NO does not stiffen the cell, suggesting that sodium-induced endothelial stiffening is likely not due to loss of NO bioavailability [61].

**Figure 3:**
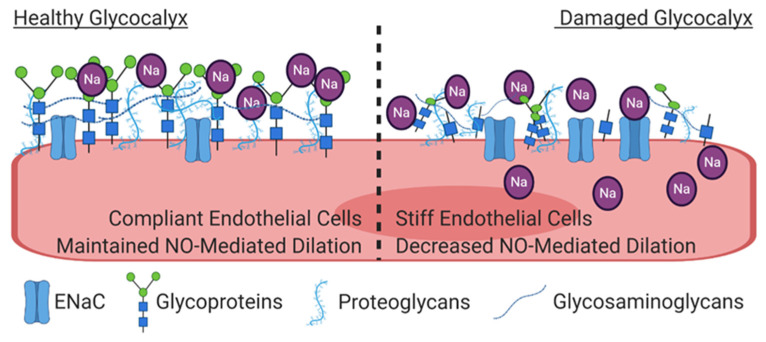
On the left, the healthy glycocalyx functions as a barrier between the plasma and the endothelial cell. The negatively charged glycocalyx buffers the positively charged sodium ions. On the right, increases in plasma sodium cause damage to the glycocalyx, thus increasing the access of sodium to epithelial sodium channels (ENaC) on the surface of the endothelium, leading to increased intracellular sodium concentrations. Under these conditions, the cell becomes stiffer, and the transduction of shear stress into NO release is decreased.

Finally, intriguing recent evidence suggests that sodium-induced damage of the endothelial glycocalyx (eGC) may initiate endothelial cell stiffening and subsequent endothelial dysfunction, as illustrated in Figure 3. The eGC is a 0.5 to 4.5 μm thick mesh-like layer of proteoglycans, glycoproteins, and glycosaminoglycans (i.e., heparin sulfate and hyaluronic acid) on the luminal surface of endothelial cells that serves as a protective barrier between the endothelium and red blood cells, and plays an important role in mechano-transduction of shear stress [62]. Deterioration of the eGC occurs with conditions that increase the risk of CVD, including aging [63], untreated hypertension [64], diabetes [65,66], and obesity [67], and may be a consequence of oxidative stress [68]. In the context of dietary sodium, the negatively charged eGC binds the positively charged sodium ions, thus buffering increases in plasma sodium levels and providing the initial endothelial cell barrier to sodium [69]. However, when endothelial cells are exposed to 150 mmol of sodium rather than 130 mmol ex vivo, the height of their eGC is reduced in a manner consistent with heparinase-induced degradation [69]. As a result, sodium has increased access to ENaC, and intracellular sodium increases rapidly, leading to augmented endothelial stiffness [70]. The sodium-induced decrease in eGC also increases monocyte adhesion and induces local inflammation [71], thus initiating further endothelial dysfunction.

## 5. Potential Countermeasures Against High Dietary Sodium-Induced Endothelial Dysfunction

Population-wide sodium reduction is proposed as the best means to mitigate the consequences of high-sodium consumption for both salt-resistant and salt-sensitive individuals [72]. For instance, by instituting policies aimed at decreasing sodium intake, Finland has reduced sodium consumption by ~40% since 1980, which contributed to an 80% reduction in the middle-aged death rate from strokes and coronary artery disease, despite increases in smoking and obesity [73]. Similarly, the sodium reduction plan instituted by the United Kingdom has decreased sodium intake by 15%, and is estimated to have prevented 9000 deaths per year [72]. However, as the sodium intake in the United States has been stable since the middle of the twentieth century [74] and a large portion of the population has no interest in reducing their sodium consumption [75], it is crucial that scientists identify non-sodium centered lifestyle changes that may help to counteract the consequences of excess sodium intake. Aerobic exercise and/or increased dietary potassium intake may diminish the vascular effects of excess sodium. If so, these behaviors could be targets for public health interventions, especially in populations that tend to resist sodium reduction.

### 5.1. Aerobic Exercise

Physical activity, or aerobic exercise in particular, is associated with reduced cardiovascular morbidity [76] and mortality [77] in adults. However, only approximately 60% of this reduction can be attributed to the effect of exercise on traditional CVD risk factors [78], suggesting non-traditional risk factors, such as endothelial function, may account for some of the remaining benefits of exercise [79]. Indeed, exercise appears to improve vascular function beyond vessels supplying the working muscles [80,81,82,83], though some controversy on this topic still exists [84]. Thus, regular physical activity may protect the vasculature from the effects of a high-sodium diet. Rebholz and colleagues report that the prevalence of salt sensitivity of BP is lowest among those in the highest quartile of physical activity [85]. While this study did not address vascular function directly, it suggests that habitual physical activity level mitigates the effects of sodium in humans; however, there is a paucity of research on this topic.

Our group recently published a report on the effects of voluntary wheel running on endothelial function in Sprague Dawley rats fed either a high or low-sodium diet for six weeks [55]. Consistent with previous reports, the high-sodium diet attenuated ACh-induced endothelium-dependent relaxation of the femoral artery, despite no group difference in BP. However, the effect of sodium on ACh-mediated relaxation was abolished in the rats who had access to a running wheel. Much of the systemic benefit of exercise on endothelial function is thought to be due to the effect of increased shear and circumferential stress on NO production (reviewed in [86]). In agreement with this hypothesis, Guers et al. showed that physical activity reversed high-sodium diet induced decreases in the ratio of phosphorylated eNOS relative to total eNOS expression. Additionally, voluntary wheel running abolished the high-sodium diet-associated increases in the expression of NADPH oxidase subunits and the decrease in expression of SOD-1. These findings support the hypothesis that regular aerobic exercise may protect against the adverse vascular effects of excess sodium consumption.

While the study by Guers et al. was not designed to determine the mechanism responsible for the beneficial effects of exercise, we can speculate on a few potential possibilities. Exercise elicits a marked increase in angiotensin II (Ang II) [87]. Ang II is a potent vasoconstrictor, and high concentrations are known to activate NADPH oxidase generation of O_2_^−^ [88]. Yet, increased sodium intake significantly suppresses plasma Ang II levels. Interestingly, when Ang II is chronically infused in animals fed high-sodium diets to restore physiological levels, endothelium-dependent relaxation is restored [57,58,89,90], O_2_^−^ production is decreased [89], and the sodium-induced decrease in SOD is abolished [57,58]. Together, this suggests that exercise may exert its vascular benefits via increases in circulating Ang II. However, this explanation is unlikely, as the half-life of Ang II in the blood is approximately 30 s [91], whereas the positive effect of subpressor doses of Ang II in animals occurs with chronic infusions.

Another possible mechanism for the endothelium-protective effects of exercise in high salt fed rats is the activation of nuclear factor erythroid 2-related factor 2 (NRF2). NRF2 is a transcription factor activated by exercise-induced ROS that, in turn, regulates expression of antioxidants and other cyto-protective genes [92,93,94]. Recently, Priestly and colleagues demonstrated that the herbal supplement Protandim, previously shown to activate NRF2 [95] and upregulate endogenous antioxidant enzymes [96], corrected sodium-induced reductions in ACh-mediated relaxation in both rat cerebral and mesenteric arteries, as well as in hamster cheek pouch arterioles [97]. Importantly, these animals exhibited increased SOD and decreased mitochondrial ROS, yet there was no effect in the NRF2^(−/−)^ knockout rats, signifying that Protandim worked specifically on NRF2 [97]. Further work is needed to determine if NRF2 activation via exercise is beneficial to rats and humans consuming a high-sodium diet.

Aerobic exercise may also protect the endothelium from excessive sodium intake by maintaining the eGC. While not studied in the context of sodium consumption, exposure to fluid shear stress in cultured endothelial cells results in a thicker eGC [98], consistent with a beneficial effect of increased blood flow, like that seen in exercise. Indeed, 20 weeks of moderate intensity aerobic training decreases markers of eGC shedding in young men [99]. Likewise, a four-week high-intensity interval training program resulted in increased eGC thickness in the sublingual microvasculature of healthy young adults and an increase in circulating microRNAs associated with eGC thickness [100]. Theoretically, such protection could prevent dietary sodium-induced eGC damage and subsequent endothelial dysfunction. A recent modelling study suggests that greater blood flow, like that occurring during exercise, reduces the number of sodium ions that bind to the eGC, thus allowing the eGC to maintain its sodium buffering capacity and mitigate the effect of sodium on endothelial function [101]. As yet, however, the potential for aerobic exercise to protect the eGC from high-sodium diets has not been experimentally tested in humans.

### 5.2. Dietary Potassium Intake

Increased dietary intake of potassium is associated with reduced risk of cardiovascular morbidity and mortality [102]. Some of the beneficial effects of potassium may come from its positive influence on endothelial function. For example, the blunted ACh-induced dilation in stroke-prone spontaneously hypertensive rats is completely prevented by eight weeks of a high-potassium diet, despite no effect on BP [103]. Similarly, 2500 mg/day of potassium supplementation in hypertensive humans who typically consumed ~3000 mg/day increased brachial artery FMD without a clinically significant reduction in BP [104]. This effect also occurred when increased potassium (5690 vs. 3110 mg/day) was consumed via food sources [105], although this benefit to endothelial function was not observed with a more moderate increase in dietary potassium (780–1560 mg/day) in individuals who typically consume ~2300 mg/day [106].

Raij and colleagues demonstrated that the addition of 3.6% potassium citrate to a high-sodium diet (8% sodium chloride) mitigated sodium-induced endothelial dysfunction in Dahl salt-sensitive rats, independent of any effect on BP, providing support for the use of potassium to combat the vascular consequences of excess dietary sodium [107]. Sundhir and colleagues also observed a protective effect of potassium supplementation (2.1% potassium chloride or 2.1% potassium bicarbonate) against the vascular insult of sodium in Dahl rats; however, these findings appeared to be related to a direct effect of potassium on systolic BP [108]. Notably, in young, salt-resistant humans, a high-potassium diet (4700 mg/day) abolished the reduction in brachial artery FMD caused by a seven-day high-sodium diet with moderate potassium intake (2500 mg/day), despite no change in either laboratory-measured or 24-hr ambulatory BP [38]. Furthermore, added dietary potassium counteracts an acute sodium insult without changing BP, as the postprandial reduction in FMD following a single high-sodium meal containing only 117 mg of potassium was completely abolished when the high-sodium meal contained 1482 mg of potassium [43]. Taken together, these findings suggest that increased consumption of potassium may be a relatively simple means to protect the vascular endothelium from a high-sodium diet.

The mechanisms through which high-potassium diets may confer vascular protection against excess dietary sodium have not been fully elucidated, but it appears potassium plays a role in modulating oxidative stress. For instance, McCabe et al. demonstrated that cultured endothelial cells and monocytes produced less ROS as the potassium content of the culture media increased [109]. Likewise, rabbits fed a low-potassium diet exhibited increased O_2_^−^ production and decreased endothelium-dependent dilation [110]. Furthermore, high-potassium feeding in Dahl salt-sensitive rats abolished high sodium induced increases in vascular O_2_^−^ and NADPH oxidase mRNA expression [111]. In addition to its effects on oxidative stress, potassium appears to regulate the deformability of endothelial cells, thus influencing how they respond to shear stress. Oberleithner and colleagues demonstrated that an increase in extracellular potassium concentration softens endothelial cells and increases NO release in response to shear stress [112]; however, more research is needed to determine the precise mechanisms behind this effect.

## 6. Conclusions

Consumption of excess dietary sodium increases the risk for CVD-related morbidity and mortality. While the processes through which dietary sodium promotes CVD are likely multifactorial, mounting evidences suggests that endothelial dysfunction may be an early contributor to this risk, as it is regularly observed as a consequence of high-sodium diets. Importantly, sodium impairs endothelial functioning in healthy individuals who do not have salt-sensitive BP, in addition to those for whom sodium increases BP. Extensive work in cell culture and animal models has been performed to examine the mechanisms through which dietary sodium harms the endothelium. This preclinical evidence suggests roles for increased oxidative stress and damage to the endothelial glycocalyx. Translation of these mechanistic studies to humans is ongoing, and will inform public health and clinical strategies to mitigate the effects of dietary sodium.

Ideally, all adults should meet the current recommendation of 2300 mg or less of sodium per day to decrease risk of CVD. However, excess sodium consumption remains ubiquitous, despite decades of attempts at reducing sodium intake. As such, additional approaches to lessen the impact of dietary sodium consumption must be explored. Aerobic exercise and increased dietary potassium consumption, for example, each directly benefit the vascular endothelium, and may protect it in the face of a high-sodium diet. More research is needed to determine if these non-sodium focused interventions are effective at mitigating the deleterious effects of dietary sodium on the vasculature, and whether these effects translate into a decrease in the burden of CVD.

## Figures and Tables

**Table 1:** Summary table of studies that have investigated the effects of dietary sodium on endothelial function.

		Sodium Intervention				
Study	Subject Characteristics	LS Content	HS Content	Duration	Endothelial Assessment	Relevant Findings
Alba et al., 2020 [27]	65 ± 7 yr *n* = 5 M/6 W Pre-Hypertensive Salt Resistant BP	1500 mg/day	5500 mg/day	Eight days	Conduit artery: FMD Microvascular: Cutaneous ACh microdialysis	HS impaired ACh-induced cutaneous dilation during a low dairy diet compared to a high dairy diet. ACh-induced vasodialtion was restored with infusion of ascorbic acid, apocynin, and Tempol. There was no effect of HS on FMD.
Baric et al., 2019 [29]	21 ± 2 yr *n*= 23M/25W Normotensive Salt Resistant BP	<1380 mg/day	~5800 mg/day (4610 mg added to normal diet)	Seven days	Microvascular: Cutaneous ACh iontophoresis	HS blunted ACh-induced cutaneous dilation.
Baric et al., 2020 [28]	20 ± 2 yr *n* = 25 M/26 W Normotensive Salt Resistant BP	<1380 mg/day	~5800 mg/day (4610 mg added to normal diet)	Seven Days	Microvascular: Cutaneous ACh iontophoresis	ACh-induced dilation was reduced by HS compared to LS in the control group, but this decrement did not occur in the group that also supplemented with vitamin C and E.
Blanch et al., 2015 [43]	37 ± 15 yr *n* = 21 M/18 W Normotensive	138 mg	1495 mg	Single Meal	Conduit Artery: FMD	FMD was reduced following the HS + low potassium meal, but not after the HS + high-potassium condition.
Cavka et al., 2016 [30]	28 ± 7 yr *n* = 12 W Normotensive	N/A	~4300 mg/day (2364 mg added to normal diet)	Seven days	Conduit Artery: FMD Microvascular: Ex-vivo Gluteal Fat Arteriole ACh and FMD	HS diet reduced brachial artery FMD from baseline. ACh- and flow-induced dilation of gluteal fat arterioles was maintained during HS diet.
Dickinson et al., 2011 [40]	37 ± 18 yr *n* = 6 M/10 W Normotensive	115 mg	1495 mg	Single Meal	Conduit Artery: FMD	FMD decreased for at least 120 min after both LS and HS meals. The reduction in FMD in the first hour after the meal was augmented with HS. Reactive hyperemia index was unchanged following either meal.
DuPont et al., 2013 [37]	33 ± 7 yr *n* = 9 M/5 W Normotensive Salt Resistant BP	460 mg/day	~7500 mg/day	Seven days	Conduit artery: FMD	Brachial artery FMD was significantly reduced following HS.
Greaney et al., 2012 [26]	31 ± 7 yr *n* = 5 M/7 W Normotensive Salt Resistant BP	460 mg/day	~7500 mg/day	Seven days	Microvascular: Cutaneous local heating	Local heating-induced cutaneous dilation was reduced following HS. Local ascorbic acid treatment augmented dilation in the HS condition.
Higashi et al., 2001 [35]	52 ± 16 yr *n* = 17 M/12 W Hypertensive Salt-sensitive and Salt Resistant BP	1150 mg/day	7820 mg/day	Seven days	Microvascular: Forearm blood flow w/ACh	ACh-induced dilation was not unaffected by dietary sodium in participants with both salt-sensitive and salt resistant BP.
Jablonski et al., 2013 [7]	62 ± 7 yr *n* = 11 M/6 W Prehypertensive or Stage 1 hypertension	~1300 mg/day	~3100 mg/day (habitual intake)	Four weeks	Conduit Artery: FMD Microvascular: Forearm blood flow w/ACh	Compared to normal sodium intake (HS), LS improved both FMD and forearm blood flow responses to ACh. FMD was improved in HS with ascorbic acid and BH4 treatment. Ascorbic acid also restored the microvascular response to ACh. Sodium-induced changes in BP were accounted for statistically.
Lennon-Edwards et al., 2014 [39]	30 ± 8 yr *n* = 16 M/14 W Normotensive, Salt Resistant	460 mg/day	~7500 mg/day	Seven days	Conduit Artery: FMD	FMD was decreased by HS in both men and women, but the decrement was greater in men.
Migdal et al., 2020 [41]	25 ± 5 yr, *n* = 17 M/20 W Normotensive	138 mg	1495 mg	Single Meal	Conduit Artery: FMD	FMD, assessed 50 min postprandial, was unchanged after either meal.
Miyoshi et al., 1997 [34]	46 ± 12 yr *n* = 15 M Hypertensive Salt-sensitive and Salt Resistant BP	1970 mg/day	7880 mg/day	Seven days	Microvascular: Forearm blood flow w/ACh	ACh-induced dilation was blunted in subjects with salt-sensitive BP versus salt resistant subjects. There was no effect of HS on ACh responses in the salt resistant group, but an increase in ACh-induced dilation occurred in the salt-sensitive group during HS.
Ramick et al., 2019 [44]	34 ± 11 yr *n* = 18 M/11 F Normotensive Salt Resistant BP	460 mg/day	6900 mg/day	Seven days	Microvascular: Cutaneous local heating	Local heating-induced dilation was impaired in the HS condition compared to LS. Local infusion of ascorbic acid, tempol, and apocynin restored microvasculature function during HS.
Smiljanec et al., 2020 [38]	27 ± 6 yr *n* = 16 M/17 W Normotensive Salt Resistant BP	1150 mg/day	6900 mg/day	Seven days	Conduit Artery: FMD	When HS was accompanied by moderate intake of potassium, FMD was reduced relative to the LS condition. High-potassium intake abolished the effects of HS on FMD.
Smiljanec et al., 2020 [42]	24 ± 6 yr *n* = 20 M/21 W Normotensive	N/A	1445 mg	Single Meal	Conduit Artery: FMD	FMD was unchanged from baseline at 60 and 120 min post-meal in both the oral antioxidant cocktail and placebo conditions.
Stein et al., 1995 [33]	34 ± 7 yr *n* = 7 M Normotensive Salt Resistant BP	230 mg/day	5750 mg/day	Five days	Microvascular: Forearm blood flow w/MCh	MCh-induced dilation was similar in HS and LS.
Tzemos, et al., 2008 [36]	25 ± 8 yr *n* = 16 M Normotensive	<920 mg/day	~5500 mg/day (4620 mg added to LS)	Five days	Microvascular: Forearm blood flow w/ACh	Forearm blood flow responses to ACh were blunted in the HS condition relative to LS. Notably, systolic BP was increased in HS, suggesting that some subjects had salt-sensitive BP.

Subject age is presented as mean ± standard deviation (SD) (calculated from published *n* and standard error where necessary). ACh: acetylcholine, BP: blood pressure, FMD: flow-mediated dilation, HS: high sodium intervention, LS: low sodium intervention, M: men, MCh: methacholine, W: women.

## Data Availability

Not applicable.
